# Periprothetischer Infekt nach Hüftprothesenimplantation: Risikofaktoren für die Entwicklung einer Frühinfektion nach Primärimplantation

**DOI:** 10.1007/s00132-022-04279-w

**Published:** 2022-07-07

**Authors:** J. Dietz, Anne Zeidler, Andreas Wienke, Alexander Zeh, Karl-Stefan Delank, David Wohlrab

**Affiliations:** 1 Departement für Orthopädie, Unfall- und Wiederherstellungschirurgie, Abteilung Endoprothetik, Universitätsklinikum Halle (Saale), Ernst-Grube-Straße 40, 06120 Halle (Saale), Deutschland; 2 Medizinische Klinik, Klinikum Obergöltzsch Rodewisch, Rodewisch, Deutschland; 3 Institut für Medizinische Epidemiologie, Biometrie und Informatik, Universitätsklinikum Halle (Saale), Halle (Saale), Deutschland

**Keywords:** Body-Mass-Index, Fall-Kontroll-Studie, C‑reaktives Protein, Retrospektive Studie, Totaler Hüftgelenkersatz, Body mass index, Case control studies, C‑reactive protein, Retrospective study, Total hip arthroplasty

## Abstract

**Hintergrund:**

Die periprothetische Infektion zählt zu den schwerwiegendsten Komplikationen in der Primärendoprothetik. Die in der aktuellen Literatur angegebenen Infektionsraten reichen von 0,36 bis 2,23 %.

**Fragestellung:**

Ziel dieser retrospektiven Fall-Kontroll-Studie war die Ermittlung von präoperativen Risikofaktoren für das Auftreten eines periprothetischen Frühinfektes nach primärer Hüftprothesenimplantation.

**Material und Methoden:**

Es wurden die Einflüsse von Patientenalter, Geschlecht, Body-Mass-Index (BMI), C‑reaktivem Protein, präoperativen Leukozytenwerten und Morbiditätsgrad (American Society of Anaesthesiologists Score) auf das Auftreten einer periprothetischen Frühinfektion am Hüftgelenk geprüft sowie deren Zusammenhang untersucht.

**Ergebnisse:**

Von den 1383 nachuntersuchten Patienten wurde bei 25 Patienten ein Frühinfekt diagnostiziert. Mit Steigerung des BMI um 1 kg/m^2^ steigt das Risiko eines periprothetischen Frühinfektes um 12,1 % (*p* < 0,001). Zudem wird mittels „receiver operating characteristic“(ROC)-Kurve ein BMI ≥ 29 kg/m^2^ als signifikanter Cut-off für die erhöhte Wahrscheinlichkeit einer periprothetischen Hüftgelenkinfektion ermittelt.

Mittels ROC-Kurve kann ein präoperativer CrP-Wert > 5 mg/l als Cut-off-Wert für ein erhöhtes Risiko für eine Frühinfektion validiert werden. Mittels binär logistischer Regression wurde statistisch kein Einfluss des CrP > 5 mg/l auf die Entstehung eines Frühinfektes nachgewiesen (*p* = 0,065).

**Diskussion:**

Patienten mit einem BMI ≥ 29 kg/m^2^ sollten auf das erhöhte Risiko einer periprothetischen Frühinfektion nach Hüftprothesenimplantation hingewiesen und es sollte eine Risikoabschätzung durchgeführt werden. Weiterhin sollte die Bestimmung des präoperativen CrP-Wertes als Standard gelten.

## Abstract (en)

**Background:**

Periprosthetic infection is one of the most serious complications in primary arthroplasty. The infection rates reported in the current literature range from 0.36 to 2.23%.

**Objectives:**

The aim of this retrospective case-control study was to determine preoperative risk factors for the occurrence of early periprosthetic infection after primary hip arthroplasty.

**Materials and methods:**

The influence of patient age, gender, body mass index (BMI), C‑reactive protein, preoperative leukocyte count and morbidity level (American Society of Anaesthesiologists score) on the occurrence of periprosthetic early infection of the hip joint was examined, and their correlation was investigated.

**Results:**

Of the 1383 patients followed up, 25 were diagnosed with early infection. With an increase in BMI of 1 kg/m^2^, the risk of periprosthetic early infection increased by 12.1% (*p* < 0.001). In addition, using the receiver operating characteristic (ROC) curve, a BMI ≥ 29 kg/m^2^ is identified as a significant cut-off for the increased likelihood of periprosthetic hip infection. Using the ROC curve, a preoperative CrP value > 5 mg/l can be validated as a cut-off value for an increased risk of early infection. Using binary logistic regression, no influence of CrP > 5 mg/l on the development of early infection was statistically proven (*p* = 0.065).

**Conclusions:**

Patients with a BMI ≥ 29 kg/m^2^ should be informed about the increased risk of periprosthetic early infection after hip prosthesis implantation and a risk assessment should be performed. Furthermore, the determination of the preoperative CrP value should be considered standard.

## Hinführung zum Thema

Die periprothetische Infektion zählt zu den schwerwiegendsten Komplikationen in der Primärendoprothetik. Jedes Jahr werden in Deutschland circa 250 primäre Hüfttotalendoprothesen pro 100.000 Einwohner implantiert. Die anhaltend hohe Zahl der Hüftendoprothesenimplantationen erfordert heute mehr denn je große Anstrengungen, um die Risiken einer periprothetischen Infektion zu minimieren. Um eine Risikoreduktion der periprothetischen Infektionen zu erzielen, ist u. a. die Bestimmung von relevanten präoperativen Risikofaktoren notwendig.

## Hintergrund und Fragestellung

Die Implantation einer Hüfttotalendoprothese zur Aufrechterhaltung der geforderten hohen Lebensqualität eines immer älter werdenden Patientenkollektivs ist oft unvermeidlich. Vor einer solchen Operation ist es die Pflicht des Operateurs, den Patienten ausreichend über Risiken und mögliche Folgen aufzuklären. Als mögliche Komplikation ist die periprothetische Gelenkinfektion zu nennen, deren Rate derzeit in der Literatur mit 0,36–2,23 % angegeben wird [12, 14]. Endoprotheseninfektionen stellen trotz jahrelanger Erfahrungen und moderner Konzepte anhaltend eine schwerwiegende Komplikation dar und führen bei den Patienten zu einem hohen Leidensdruck, aufgrund von langen Krankenhausaufenthalten mit sozialer Isolation sowie funktionellen Einschränkungen und Schmerzen. Des Weiteren bedingen sie für die Sozialkassen hohe Kosten durch die langwierigen Krankenhausaufenthalte, anschließende Rehabilitationsmaßahmen oder den Ausfall der Arbeitstätigkeit. Daher ist es notwendig, die Rate periprothetischer Infektionen am Hüftgelenk zu reduzieren. In zahlreichen Publikationen konnten bereits Diabetes mellitus, Adipositas, eine Immunsuppression und maligne Erkrankungen als Risikofaktoren benannt werden [2, 13]. Es ist jedoch notwendig, konkrete Risikofaktoren zu benennen, damit diese präoperativ optimiert werden können.

Ziel der vorliegenden Studie war es, praktikable und verlässliche Parameter zu definieren, die Einfluss auf das Auftreten eines Frühinfektes nach primärer totalendoprothetischer Versorgung des Hüftgelenkes haben können. Hierzu wurde untersucht, ob Patientenalter, Geschlecht, Body-Mass-Index (BMI), präoperativer Wert des C‑reaktiven-Proteins (CrP-Wert), präoperative Leukozytenzahl und Morbiditätsgrad der Patienten (gemessen mithilfe des American Society of Anesthesiologists-Scores [ASA-Score]) und die Abhängigkeit der Parameter zueinander das Risiko für einen späteren Frühinfekt nach Hüftendoprothesenimplantation beeinflussen. Außerdem sollten bisherige Literaturergebnisse auf eine Übertragbarkeit auf unser Patientenkollektiv überprüft werden.

## Studiendesign und Untersuchungsmethoden

Es wurden retrospektive Daten von insgesamt 1383 Patienten, die am Universitätsklinikum Halle (Saale) eine primäre Hüftendoprothese erhielten, aus den Patientenakten entnommen. 25 Patienten erlitten eine periprothetische Infektion der Hüfttotalendoprothese in Sinne eines Frühinfektes. Als Frühinfektion wurde eine Infektion, die innerhalb von 4 Wochen nach Primärimplantation auftrat, definiert. Patienten mit später auftretenden periprothetischen Infektionen oder aseptischen Revisionsoperationen wurden aus der Studie ausgeschlossen. Dementsprechend konnten die Patienten der Infektgruppe (*n* = 25) oder der Kontrollgruppe (*n* = 1358) zugeordnet werden.

Aus den Patientenakten wurden als relevante präoperative Daten das Geschlecht und das Geburts- sowie Operationsdatum zur Berechnung des Alters entnommen. Körpergröße und -gewicht dienten der Ermittlung des Body-Mass-Index. Ziel war es, zu untersuchen, ob ein Cut-off-Wert des Body-Mass-Index festgelegt werden kann, ab dem das Risiko für einen periprothetischen Frühinfekt nach Hüfttotalendoprothesenimplantation steigt. Als wesentliche Laborparameter wurde der Einfluss des C‑reaktiven Proteins und der präoperativen Leukozytenanzahl bezüglich einer Frühinfektion untersucht. Von besonderem Interesse war, ob der Cut-off-Wert für das CrP von 5 mg/l oder 0,5 mg/dl, welcher von Pfitzner et al. publiziert wurde, auch für das vorliegende Patientenkollektiv validiert werden kann [10]. Zur Beurteilung der Morbidität erfolgte abschließend die Einbeziehung des ASA-Scores aus dem Anästhesieprotokoll.

Zunächst wurde untersucht, inwiefern sich die relevanten Prädiktoren in Infekt- und Kontrollgruppe unterscheiden. Dazu wurde der Chi-Quadrat-Test für nominale Variablen mit Zellenhäufigkeiten über fünf angewendet. Für Zellenhäufigkeiten kleiner fünf wurde der exakte Fischer-Test durchgeführt. Der Mann-Whitney-U-Test kam für nicht normalverteilte metrisch und ordinal skalierte Einflussvariablen zum Einsatz. Die metrischen Variablen wurden zusätzlich gruppiert untersucht. Mittels binär logistischer Regression sollte überprüft werden, in welchem Maße die Prädiktoren Einfluss auf die Frühinfektion ausüben, zunächst für jeden Prädiktor einzeln, anschließend alle Prädiktoren unter Verwendung der Einschlussmethode. Als Signifikanzniveau wurde *p* < 0,05 festgelegt. Die statistische Auswertung der Daten erfolgte mit dem Statistikprogramm SPSS Version 21.0 ® (IBM, Amonk, NY, USA).

## Ergebnisse

### Demografische Daten

Als möglicher Einflussfaktor für die Entwicklung eines periprothetischen Frühinfektes am Hüftgelenk wurde das Patientengeschlecht untersucht. Es wurden 710 Frauen (51 %) und 673 Männer (49 %) eingeschlossen. 56 % der Patienten mit einem periprothetischen Frühinfekt waren männlich. Dennoch zeigte das Geschlecht keinen Zusammenhang mit dem Auftreten einer periprothetischen Frühinfektion nach Hüfttotalendoprothesenversorgung (*p* = 0,46). Patienten der Kontrollgruppe (64,39 ± 12,03 Jahre) waren zur Primäroperation durchschnittlich 3,5 Jahre älter als Patienten der Infektgruppe (60,72 ± 12,09 Jahre). Der Prädiktor Alter konnte in der vorliegenden Studie statistisch nicht gegen den Zufall abgesichert werden (*p* = 0,131). Weiterhin wurde untersucht, ob Patienten, die zur Primäroperation älter als 80 Jahre waren, ein erhöhtes Risiko für eine periprothetische Infektionen aufweisen. Diesbezüglich konnte kein Einfluss festgestellt werden (*p* = 0,57).

### Body-Mass-Index (BMI)

**Figure 1:**
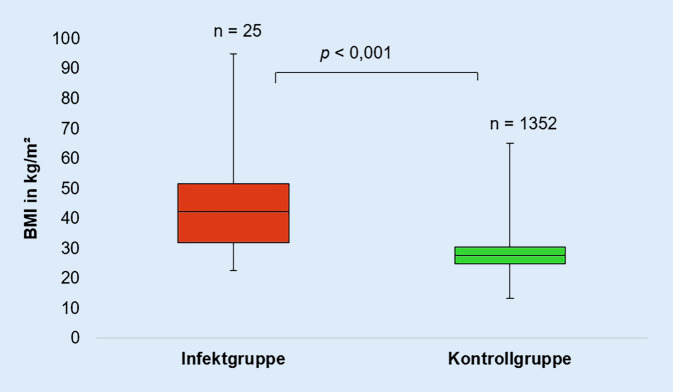

**Figure 2:**
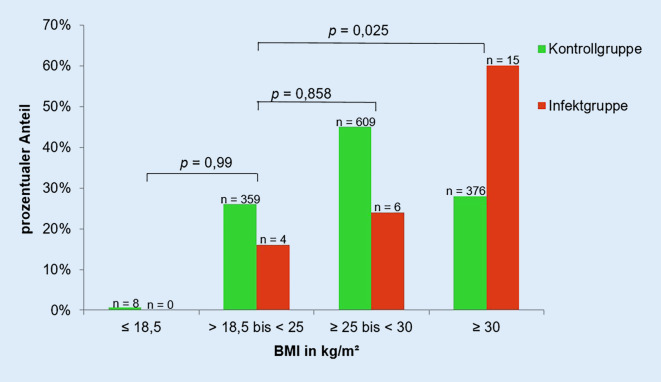

**Figure 3:**
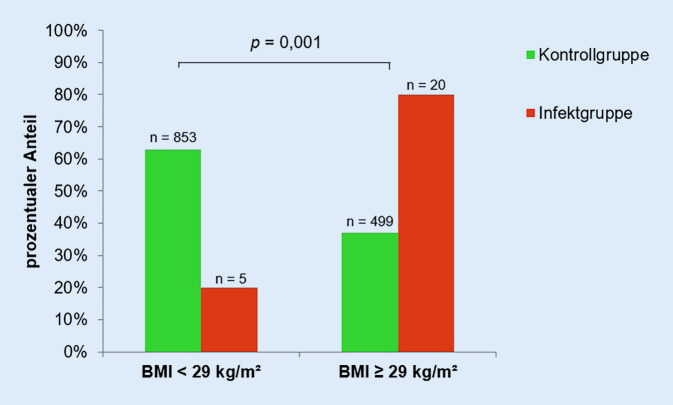

Der aus Körpergröße und -gewicht ermittelte BMI betrug im Durchschnitt 31,6 (±5,42) kg/m^2^ bei der Infektgruppe und 28,0 (±4,7) kg/m^2^ bei der Kontrollgruppe (Abb. 1). Der BMI hat somit einen hochsignifikanten Einfluss auf die Entstehung einer periprothetischen Frühinfektion des Hüftgelenks (*p* < 0,001). Mit Steigerung des BMI um eine Einheit (= 1 kg/m^2^) stieg die Wahrscheinlichkeit einen periprothetischen Frühinfekt des Hüftgelenkes zu erleiden um 12,1 % (*p* > 0,001, 95%iges CI 1,06–1,19). Entsprechend der WHO-Einteilung des BMI wurde sowohl die Infekt- als auch die Kontrollgruppe in Patienten mit Untergewicht (BMI < 18,5 kg/m^2^), Normalgewicht (BMI < 18,5 bis < 25 kg/m^2^), Übergewicht (BMI ≥ 25 bis < 30 kg/m^2^) und starkem Übergewicht (BMI ≥ 30 kg/m^2^) eingeteilt (Abb. 2). Der Hauptteil der Infektgruppe (*n* = 15) hatte präoperativ einen BMI ≥ 30 kg/m^2^, bei keinem Patienten der Infektgruppe bestand Untergewicht. Hieraus ist zu schlussfolgern, dass Patienten mit starkem Übergewicht gegenüber Normalgewichtigen ein höheres Risiko haben einen periprothetischen Frühinfekt zu erleiden (*p* = 0,025). Mittels „receiver operating characteristic“(ROC)-Kurve wurde ein präoperativer BMI von 29 kg/m^2^ als Cut-off-Wert bestimmt, ab dem das Risiko für einen Frühinfekt der implantierten Hüfttotalendoprothese steigt. Infekt- und Kontrollgruppe wurden anhand des ermittelten Cut-off Wertes von 29 kg/m^2^ eingeteilt (Abb. 3). Ein BMI ≥ 29 kg/m^2^ steigert, unabhängig von anderen Patientendaten, das Risiko einen periprothetischen Frühinfekt zu erleiden (*p* = 0,001) um das 4,8Fache.

### C-reaktives Protein (CrP)

Das C‑reaktive Protein stellt ein zuverlässiges Maß für eine im Körper ablaufende Entzündungsreaktion dar [5]. Patienten mit einem periprothetischen Frühinfekt (9,78 ± 5,91 mg/l) hatten präoperativ einen durchschnittlich etwa 1,6 mg/l höheren CrP-Wert als Patienten ohne diese Komplikation (8,13 ± 17,21 mg/l). Dieser deskriptiv beschriebene Unterschied zeigte in dem von uns untersuchten Patientenkollektiv statistisch keinen Zusammenhang (*p* = 0,059) mit dem Auftreten einer periprothetischen Frühinfektion.

**Figure 4:**
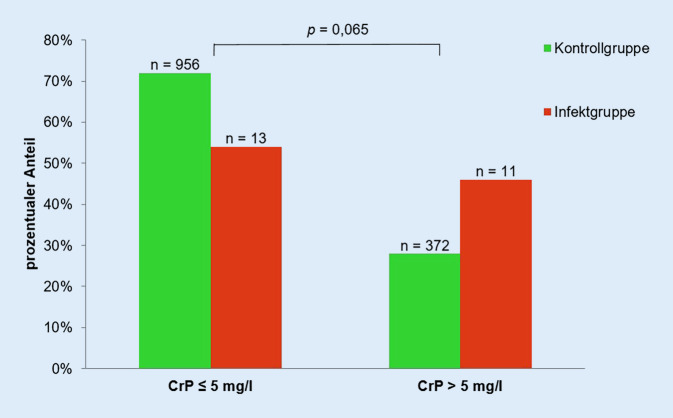

Mittels ROC-Kurve wurde anhand der vorliegenden Patientendaten untersucht, ab welchem präoperativen CrP-Wert das Risiko einer periprothetischen Frühinfektion steigt. Es konnte ein CrP-Wert von 5,1 mg/l bestimmt werden, deren Zuordnung zur Infekt- bzw. Kontrollgruppe in Abb. 4 zur Darstellung kommt. Statistisch konnte kein Einfluss des CrP > 5 mg/l auf die Entstehung eines periprothetischen Frühinfektes nach Hüfttotalendoprothesenimplantation nachgewiesen werden (*p* = 0,065).

### Leukozytenzahl

Als weiterer laborchemischer Entzündungsparameter wurde der Einfluss der präoperativen Leukozytenanzahl auf die Entwicklung einer periprothetischen Frühinfektion des Hüftgelenks untersucht. Sowohl in der Infekt- als auch in der Kontrollgruppe betrug der durchschnittliche präoperative Leukozytenwert 7 Gpt/l. Zwischen den Gruppen zeigte sich kein Unterschied, sodass ein Einfluss auf das Auftreten eines periprothetischen Frühinfektes im untersuchten Patientenkollektiv nicht gezeigt werden konnte (*p* = 1,0). Auch nach der Aufteilung der Infekt- und Kontrollgruppe in Patienten mit einer präoperativen Leukopenie, normwertigen Leukozytenanzahl und Leukozytose bestand in der geclusterten Form kein Zusammenhang (*p* = 0,357) zwischen der präoperativen Leukozytenanzahl und dem Auftreten eines periprothetischen Frühinfektes.

### ASA-Score

**Figure 5:**
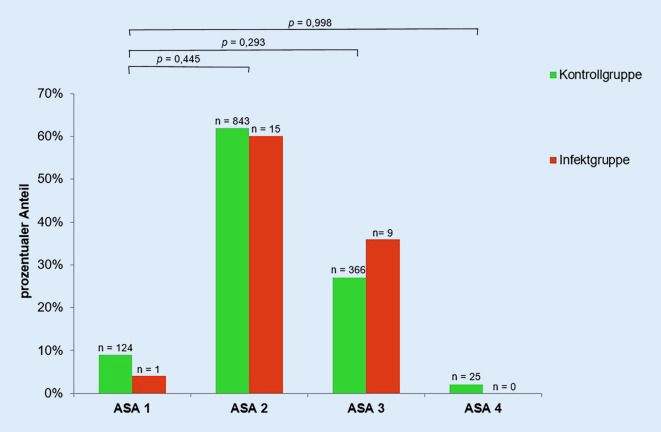

Die ASA-Klassifikation wurde von der American Society of Anaesthesiologists erarbeitet und klassifiziert den physischen Status eines Patienten. Der ASA-Score lässt somit Rückschlüsse auf die Morbidität zu. Die ASA-Score-Verteilung von Infekt- und Kontrollgruppe spiegelt Abb. 5 wider. Kein Patient der Infektgruppe hatte einen ASA-Score von 4. Es konnte nicht bestätigt werden, dass mit zunehmendem ASA-Wert das Risiko einer periprothetischen Hüftgelenkinfektion steigt. 36 % der Patienten der Infektgruppe und 29 % der Patienten der Kontrollgruppe wiesen einen ASA-Score über 2 auf. Statistisch konnte kein Zusammenhang zwischen einem ASA-Wert > 2 und dem Auftreten eines Frühinfektes nach endoprothetischer Versorgung des Hüftgelenks nachgewiesen werden (*p* = 0,434).

### Abhängigkeit der Parameter

Die binär logistische Regression sollte Aufschluss darüber geben, ob der BMI oder der CrP-Wert – auch unter Berücksichtigung der anderen Parameter – von Bedeutung sind. In die multivariate binär logistische Regression wurden zunächst die Parameter in ihrer Grundform eingebracht (Tab. 1). Alleinig der BMI hatte einen signifikanten Einfluss auf die Entstehung eines Frühinfektes (*p* = 0,001).

**Table 1:** 

Prädiktor	*p*	Odds Ratio	95%iges Konfidenzintervall
Alter	0,4	0,96	0,93–0,997
Geschlecht	0,29	1,59	0,68–3,74
ASA 1(RG)	0,48	–	–
ASA 2	0,37	2,63	0,32–21,25
ASA 3	0,17	4,64	0,51–42,39
ASA 4	0,99	0,00	0,00
Leukozyten	0,70	0,96	0,77–1,19
CrP-Wert in mg/l	0,54	1,01	0,98–1,04
BMI in kg/m^2^	0,001	1,12	1,05–1,19

*ASA* American Society of Anaesthesiologists, *BMI* Body-Mass-Index, *CrP* C-reaktives Protein

Eine weitere Regressionsanalyse der kategorisierten Prädiktoren (Tab. 2) bestätigte ebenfalls den Einfluss des BMI auf die Entstehung eines periprothetischen Frühinfektes. Es wird deutlich, dass Patienten mit einem BMI ≥ 29 kg/m^2^ ein 6,14fach höheres Risiko haben, eine Frühinfektion zu erleiden (*p* < 0,001), wenn das Alter > 80, der CrP-Wert > 5, der ASA-Score > 2 und die Leukozytenanzahl nicht normwertig sind.

**Table 2:** 

Prädiktor	*p*	Odds Ratio	95%iges Konfidenzintervall
Alter > 80 Jahre	0,34	0,68	0,45–9,87
Geschlecht	0,35	0,68	0,29–1,55
ASA > 2	0,77	1,14	0,48–2,70
Normale Leukozyten (RG)	0,89	–	–
Leukopenie	0,99	0,00	0,00
Leukozytose	0,63	1,45	0,32–6,49
CrP > 5 mg/l	0,28	1,59	0,69–3,66
BMI 2: 29 kg/m^2^	< 0,001	6,14	2,24–16,86

*ASA* American Society of Anaesthesiologists, *BMI* Body-Mass-Index, *CrP* C‑reaktives Protein

## Diskussion

### Demografische Daten

Das Alter als Risikofaktor für die Entstehung einer periprothetischen Gelenkinfektion insbesondere des Hüftgelenks wurde in zahlreichen Studien untersucht. Ridgeway et al. und Wu et al. kamen zu dem Ergebnis, dass ältere Patienten ein erhöhtes Infektionsrisiko haben [14, 15]. Dagegen konnten Poultsides et al. oder Malinzak et al. ein erhöhtes Risiko für jüngere Patienten zeigen [7, 12]. Namba et al. kamen zu dem Ergebnis, dass vom Patientenalter kein Einfluss auf die Entstehung einer periprothetischen Hüftgelenkinfektion ausgeht [8]. Diese Aussage konnte in der vorliegenden Studie bestätigt werden. Die Ergebnisse der aktuellen Literatur geben jedoch einen unterschiedlichen Zusammenhang zwischen Patientenalter und periprothetischer Hüftgelenkinfektion wieder. Bezüglich des Patientengeschlechtes konnte in der vorliegenden Studie statistisch kein Zusammenhang mit dem Auftreten einer Gelenkinfektion nach Hüftendoprothesenversorgung nachgewiesen werden. Dieses Resultat bestätigte die Ergebnisse der Studien von Agodi et al. und Choong et al. [1, 3]. Eine Untersuchung von Ridegeway et al. konnte jedoch zeigen, dass Frauen ein erhöhtes Risiko haben [14]. Poultsides et al. hingegen wiesen nach, dass Männer ein erhöhtes Risiko für das Auftreten einer periprothetischen Hüftgelenkinfektion haben [12]. Die aktuelle Literatur ergibt bezüglich des Einflusses des Geschlechts auf die Entstehung eines periprothetischen Gelenkinfekts keinen eindeutigen Zusammenhang.

### Body-Mass-Index (BMI)

Aus Körpergröße und -gewicht wurde in der vorliegenden Studie der BMI ermittelt, welcher einen signifikanten Einfluss auf das Auftreten einer periprothetischen Gelenkinfektion (*p* = 0,001) zeigte. Auch wird deutlich, dass Patienten mit einem BMI ≥ 29 kg/m^2^ gegenüber normalgewichtigen Patienten ein höheres Risiko haben, einen Frühinfekt der implantierten Hüfttotalendoprothese zu entwickeln (*p* = 0,025). Eine anonymisierte Befragung deutscher Ärzte aus dem Jahr 2021 konnte zeigen, dass für 48 % eine Kontraindikation für die Hüftprothesenimplantation erst ab einem BMI ≥ 40 kg/m^2^ besteht und die Adipositas per magna nicht konsistent als Kontraindikation gesehen wird [11]. Die aktuelle Literatur beschreibt jedoch die Gefahr eines verlängerten stationären Aufenthaltes und erhöhter Morbidität und Mortalität aufgrund präoperativer Risikofaktoren (Diabetes, Adipositas, Anämie etc.) [13]. Ridgeway et al., Choong et al. und Lenguerrand et al. konnten zeigen, dass ein BMI > 30 kg/m^2^ mit einem signifikant erhöhten Risiko (*p* < 0,01) für eine periprothetische Hüftgelenkinfektion einhergeht [3, 6, 14]. In der vorliegenden Studie besteht mit Erhöhung des BMI um 1 kg/m^2^ ein 12,1 % höheres Risiko einen Frühinfekt zu erleiden. Peel et al. fanden heraus, dass mit jeder Steigerung des BMI um 1 kg/m^2^ das Risiko einer periprothetischen Hüftgelenkinfektion um 10 % steigt [9]. Mittels ROC-Kurve konnte ein Cut-off-Wert von 29 kg/m^2^ als signifikanter Risikofaktor für die Entstehung einer periprothetischen Hüftgelenkinfektion festgestellt werden (*p* < 0,001). Patienten mit einem BMI ≥ 29 kg/m^2^ hatten zudem ein 4,76fach höheres Risiko. Wu et al. konnten zeigen, dass ein BMI ≥ 28 kg/m^2^ mit einem 2,77fach erhöhtem Risiko einhergeht, eine periprothetische Gelenkinfektion zu erleiden als mit einem BMI zwischen 18,5 und 28 kg/m^2^ (*p* = 0,017) [15]. Die in dieser Studie ermittelten Resultate bestätigen die Ergebnisse der aktuellen Literatur bzgl. des Zusammenhanges des BMI mit dem Auftreten eines periprothetischen Infektes des Hüftgelenkes. Patienten mit einem BMI ≥ 29 kg/m^2^ sollten über das erhöhte Risiko einer periprothetischen Frühinfektion nach Hüftprothesenimplantation aufgeklärt werden. Es empfiehlt sich, präoperativ eine Risiko-Nutzen-Abschätzung durchzuführen.

### C-reaktives Protein (CrP)

In der Literatur wird ein CrP-Wert > 5 mg/l als präoperativer Marker für das Auftreten eines periprothetischen Infektes beschrieben [10]. In der vorliegenden Studie ergab sich jedoch kein signifikanter Zusammenhang zwischen einer CrP-Erhöhung und einem Frühinfekt nach Hüftendprothesenimplantation (*p* = 0,059). Als Grenzwert für ein erhöhtes Risiko, einen Frühinfekt zu erleiden, wurde mithilfe einer ROC-Kurve ein CrP > 5 mg/l ermittelt. Der Effekt auf die Entstehung eines Frühinfektes mit einem präoperativen CrP > 5 mg/l zeigte keine Signifikanz (*p* = 0,065). Die Ergebnisse von Pfitzner et al. (2008) können für das Patientenkollektiv der vorliegenden Studie übernommen werden [10]. Mit einer Wahrscheinlichkeit von 78,6 % wurden Patienten mit einem CrP > 5 mg/l als Patienten mit späterem Frühinfekt der implantierten Hüfttotalendoprothese erkannt. Die Bestimmung des präoperativen CrP-Wertes sollte als Standard gelten. Bei Patienten mit einem präoperativen CrP-Wert > 5 mg/l ist eine systemische und lokale Fokussuche zu empfehlen (körperliche Untersuchung, Urin-Kultur, Röntgen-Thorax, zahnärztliche Untersuchung). Kann kein Infektfokus gefunden werden, ist der Patient über sein erhöhtes Infektionsrisiko aufzuklären und gemeinsam eine Risikoabschätzung zu diskutieren.

### Leukozytenzahl

Bezüglich der präoperativen Leukozytenzahl konnte kein signifikanter Zusammenhang mit der Komplikation der Frühinfektion nach Endoprothesenversorgung nachgewiesen werden. Der präoperative Leukozytenwert zeigte in der vorliegenden Studie keinen Einfluss auf die Entstehung einer periprothetischen Hüftgelenkinfektion. Die Ergebnisse der Studie von Cordero-Ampuero und Dios bezüglich der Leukozytenzahl können für unser Patientenkollektiv bestätigt werden [4].

### ASA-Score

Der ASA-Wert als Maß für die Patientenmorbidität und auch die Untersuchung des ASA > 2 zeigten im untersuchten Patientenkollektiv keinen Einfluss auf die Entstehung einer periprothetischen Frühinfektion des Hüftgelenks. Auch Debreuve-Theresette et al. konnten keinen Einfluss des ASA-Wertes auf die Entwicklung einer periprothetischen Hüftgelenkinfektion feststellen [5]. Namba et al. und Lenguerrand et al. wiesen jedoch nach, dass ein ASA-Wert > 2 mit einem höheren Risiko einhergeht [6, 8]. Dennoch lässt sich der ASA-Score für den Patienten und eine fundierte Aufklärung im Besonderen über das Risiko einer periprothetischen Hüftgelenkinfektion schwierig in den Alltag übertragen. Spezifischer kann man Patienten bei Vorerkrankungen wie Diabetes mellitus, chronischen Lungenerkrankungen, Lebererkrankung, Herzinsuffizienz und rheumatologischen Erkrankungen darüber aufklären, dass ein erhöhtes Risiko für die Komplikation der periprothetischen Infektion besteht, wie es Lenguerrand et al. nachgewiesen haben [6].

### Abhängigkeit der Parameter

Die binär logistische Regression mit allen Parametern ergab, dass ausschließlich der BMI einen signifikanten Einfluss auf das Auftreten einer periprothetischen Hüftgelenkinfektion hat. Der BMI sollte damit im Fokus vor einer elektiven Operation stehen. Alter, Geschlecht, Leukozytenanzahl und ASA-Score scheinen am vorliegenden Patientenkollektiv für die Entstehung einer periprothetischen Frühinfektion nach Hüfttotalendoprothesenimplantation weniger von Bedeutung zu sein.

## Fazit für die Praxis

- Patienten mit einem Body-Mass-Index ≥ 29 kg/m^2^ gelten als Risikopatienten für die Entwicklung eines Frühinfektes nach totalendoprothetischer Versorgung des Hüftgelenkes, insbesondere, wenn sie über 80 Jahre alt und multimorbide sind und präoperativ erhöhte Entzündungswerte aufweisen.
- Auf dieses erhöhte Risiko sollte im Rahmen der Operationsaufklärung hingewiesen und eine Risikoabschätzung durchgeführt werden.
- Der präoperative CrP(C-reaktives Protein)-Wert zeigt in der vorliegenden Studie keinen signifikanten Zusammenhang mit dem Auftreten eines periprothetischen Infektes. Es scheint aber eine Übereinstimmung zu geben, dass ein CrP > 5 mg/l ein Risikofaktor für eine periprothetische Hüftgelenkinfektion darstellt. Die Bestimmung des präoperativen CrP-Wertes sollte weiterhin als Standard gelten.
- An die vorliegende Untersuchung könnten sich weitere Studien vor allem der intra- und postoperativen Risikofaktoren für eine periprothetische Infektion, auch der Spätinfektionen, anschließen.
